# An Experimental Study on the Dielectric Properties of Rubber Materials

**DOI:** 10.3390/polym13172908

**Published:** 2021-08-29

**Authors:** Hailong Chen, Yudong Xu, Mengqi Liu, Tao Li

**Affiliations:** College of Electromechanical Engineering, Qingdao University of Science & Technology, Qingdao 266061, China; xyd15621007430@163.com (Y.X.); 17852013378@163.com (M.L.); qustchl100@163.com (T.L.)

**Keywords:** rubber, dielectric properties, mixing process, carbon black

## Abstract

According to specific formulas, the mixing of rubber samples occurs by two methods: open mixing and internal mixing. The effects of frequency, mixing process, carbon black (CB) content, zinc oxide (ZnO) content, and stearic acid (SA) content on the dielectric properties of rubber materials were studied. The results showed that the effects of the mixing process on the dielectric properties of the rubber samples cannot be ignored, and the appropriate mixing process should be selected when preparing the required rubber materials. The dielectric constant and loss factor of the rubber samples vary depending on the frequency. The dielectric constant had a peak and valley value, while the loss factor only had a peak. The dielectric constant and loss factor of rubber samples were significantly affected by the content of CB, ZnO, and SA. The peak frequency decreased with the increase in CB content, however, the dielectric constant increased with an increase in CB content. The higher the ZnO content, the lower the peak frequency. In addition, the dielectric constant and loss factor increased with an increase in ZnO content. The higher the SA content, the greater the peak frequency. In addition, the dielectric constant and loss factor decreased with an increase in SA content. It is hoped that the experimental results obtained can provide guidance for the study of the dielectric properties, microwave absorption properties, and microwave heating characteristics of rubber polymers.

## 1. Introduction

Rubber materials are widely used in important fields such as military defense, aerospace, and transportation. Raw rubber must undergo a vulcanization process to obtain good performance in rubber products. Microwave vulcanization technology is favored by rubber science and technology workers due to its advantages such as fast heating rate, cleanliness, and convenient control. However, the microwave heating behavior of rubber is affected by its dielectric properties. Different dielectric properties will lead to different microwave heating temperature distribution and heating efficiency, in addition, the distribution of the microwave heating temperature is important to ensure the good performance of rubber products [1,2]. Therefore, it is extremely important to study the dielectric properties of rubber materials.

In recent years, the dielectric properties or microwave absorbing properties of rubber materials have attracted the attention of many scholars. Silicone rubber (SR) is widely used in dielectric applications due to its excellent chemical resistance, electrical insulation, and good flexibility. In order to improve the dielectric properties or absorbing properties of SR, nanofillers such as carbon nanotubes, graphene, clay, and ceramics are added to SR to prepare dielectric materials that meet the needs of rubber products [3,4,5,6,7,8,9,10,11,12,13,14,15]. Carbon nanotubes have good electrical and microwave absorbing properties, so they have attracted the attention of materials science and technology workers. Carbon nanotubes are used as fillers to improve the dielectric properties, electromagnetic shielding performance, and wave absorbing properties of polymer materials [16,17,18,19]. In addition to carbon nanotubes, graphene has also attracted the attention of some scholars due to its excellent thermal, electrical, and microwave absorbing properties, and graphene is also used as a filler to improve the dielectric properties and microwave absorbing properties of polymer materials [20,21,22]. Polymer materials need to undergo a curing process to obtain application performance. Some scholars have found that curing agents and curing or filling networks will also have a significant impact on the dielectric behavior or dielectric response of the cured polymer [23,24,25,26]. Dielectric elastomers (DE) can be used as dielectric elastomer actuators (DEAs), which can induce surprisingly large actuated strain under an electric field and thus have attracted much attention. However, in order to obtain the good performance of DEAs, the dielectric properties of polymer composites, especially the dielectric constant, are not negligible research objects [27,28].

Because tires are thick-section rubber products, traditional vulcanization methods tend to cause defects such as uneven vulcanization temperature and low vulcanization efficiency. If microwave vulcanization of large tires can be achieved, production efficiency and energy consumption can be significantly improved. Therefore, in this work, tire rubber is the research object. Firstly, rubber samples with different formulas were prepared using different mixing processes. Secondly, the dielectric constants of the rubber samples within a certain microwave frequency range were measured, and then the effects of the mixing processes on the dielectric properties of the rubber samples were studied. Finally, the effects of the CB content, ZnO content, and SA content on the dielectric constant and loss factor were studied.

## 2. Experiment

In order to carry out the experiment, firstly, the raw materials and equipment for the experiment were prepared, and then the rubber samples were prepared by the mixing processes according to the rubber mixing formulas. Finally, the complex permittivity of the prepared samples was measured by a broadband dielectric and impedance spectrometer.

The expression of complex permittivity is $\varepsilon =\varepsilon^{\prime }-\varepsilon^{\prime \prime }\times j$. $\varepsilon^{\prime }$ is the real part of the complex permittivity, also called relative permittivity (or the dielectric constant). It indicates the relative ability of a dielectric to store electrostatic energy in an electric field. $\varepsilon^{\prime \prime }$ is the imaginary part of the complex permittivity, also known as the dielectric loss factor (or the loss factor). It represents the relative ability of a dielectric to convert electrical energy into heat.

### 2.1. Materials

NR was purchased from Shanghai Yanzhong Industrial Co., Ltd., (Shanghai, China). ZnO was purchased from Shanghai Liangjiang Titanium White Chemical Co., Ltd., (Shanghai, China). SA was provided by Tianjin Beichen Founder Reagent Factory, (Tianjin, China). CB was purchased from Suzhou Kabo Fine Chemicals Co., Ltd., (Suzhou, China). In addition, sulfur (S) was provided by Fengcheng Fuhua Chemical Co., Ltd., (Fengcheng, China). The technical indicators of ZnO and CB are listed in Table 1.

### 2.2. Experimental Device

**Figure 1:**
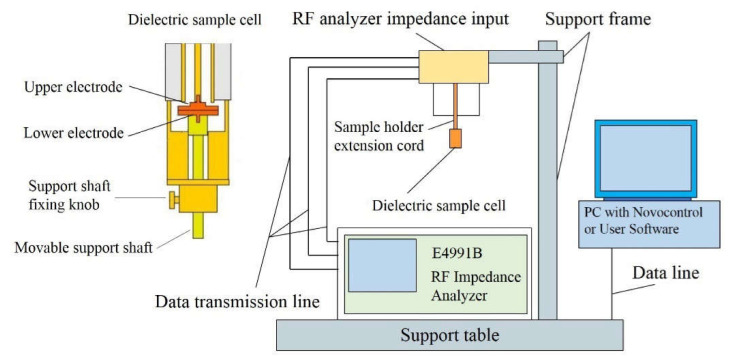
The schematic diagram of BDIS.

The XQL-160 rubber cutting machine was used to cut natural rubber blocks. The X (m)-1.7L synchronous rotor internal mixer was used for closed refining of rubber blends. The XK-160 open mill was used for open refining of the rubber blends and calendering the rubber blends into sheet samples. The dielectric properties (dielectric constant and loss factor) of the samples were measured by using broadband dielectric and impedance spectroscopy (BDIS). The schematic diagram of BDIS is shown in Figure 1. The technology parameters of the broadband dielectric and impedance spectroscopy are shown in Table 2. BDIS measurements were performed on a high resolution dielectric analyzer (Novocontrol GmbH).

**Figure 2:**
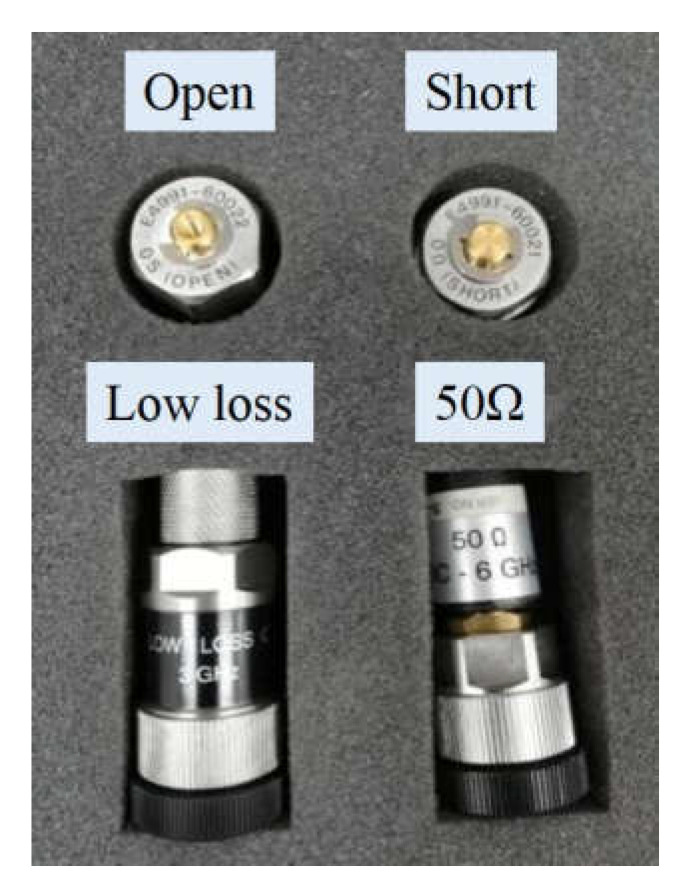
Four standard parts for calibration.

Experimental steps: (1) Calibration of four standard parts (shown in Figure 2). The four standard parts were fixed on the extension line of the sample rack in sequence and calibrated, and then the calibration results were saved. Calibration sequence: ① Open, ② Short, ③ 50 Ω, ④ Low loss. (2) Calibration of the dielectric sample cell and saving of the calibration results. The dielectric sample cell was fixed on the extension line of the sample holder, and then the prepared rubber sample was placed between the upper electrode and the lower electrode, the sample cell was sealed, and the experimental results were obtained by measurement. The experimental rubber sample was a circular film with a diameter of 11 mm and a thickness of about 1.8 mm.

### 2.3. Preparation of Rubber Samples

#### 2.3.1. The Formula for Mixing Rubber

ZnO and SA are indispensable additives for making tire rubber. ZnO can be used as a vulcanization activator to accelerate the vulcanization of natural rubber. It can not only strengthen the vulcanization process but also has the effect of strengthening and anti-aging. The amount used in the formula was about 1–5%. In the tire manufacturing process, SA can be used as an external lubricant to soften and plasticize natural rubber, and it can also be used as a vulcanization accelerator to accelerate the rubber vulcanization process. The amount used in the formula was about 0.5–3%. Therefore, in order to study the dielectric properties of rubber, the effect of the content of ZnO and SA also needed to be explored. According to the foregoing, the formulas of mixing rubber are shown in Table 3.

#### 2.3.2. The Mixing Process of the Rubber Sample

**Figure 3:**
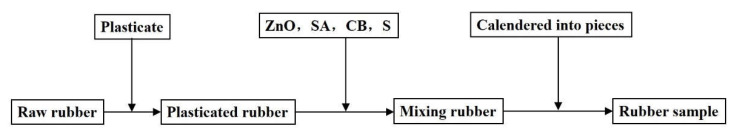
The process flow diagram of the open mixing technology.

**Figure 4:**
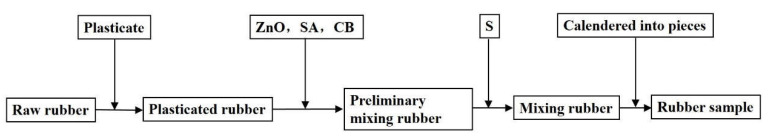
The process flow diagram of the internal mixing technology.

At present, dry mixing technology mainly includes open mixing and internal mixing. An open mill and an internal mixer are used in the two mixing methods, respectively. In order to explore whether the dielectric properties of rubber are affected by the mixing method, open mixing and internal mixing methods were selected for the preparation of natural rubber samples. For plasticizing and mixing, the natural rubber was cut into small pieces by a rubber cutter, and then the small pieces of natural rubber underwent an open mixing process and an internal mixing process to obtain rubber samples. The open mixing conditions and internal mixing conditions are listed in Table 4. The mixing process flows are shown in Figure 3 and Figure 4. In the open mixing process, all the processes were completed by the open mill. These processes included the plasticizing of natural rubber, the uniform mixing of the plasticized rubber and additives, and the calendering of the rubber blends. However, the preparation of the internal mixing rubber sample was not entirely completed by the internal mixer. The main reason was that the temperature of the internal mixing process can reach 130 °C or more, and the vulcanization reaction of the natural rubber and S will cause the rubber to crosslink. Therefore, S could not be added to the internal mixer, and the mixing of S and the natural rubber were completed by the open mixer. No matter what kind of mixing method, all the rubber blends must be calendered by an open mill. The calendered rubber sheet was cut into a circular test sample with a diameter of 11 mm for the experimental measurement of dielectric properties.

## 3. Results and Discussion

### 3.1. Effect of the Mixing Process

According to the rubber mixing formula four, the rubber samples containing different mass CB were prepared by using the open mill and internal mixer. The codes of the experimental rubber samples corresponding to formula four are shown in Table 5.

**Figure 5:**
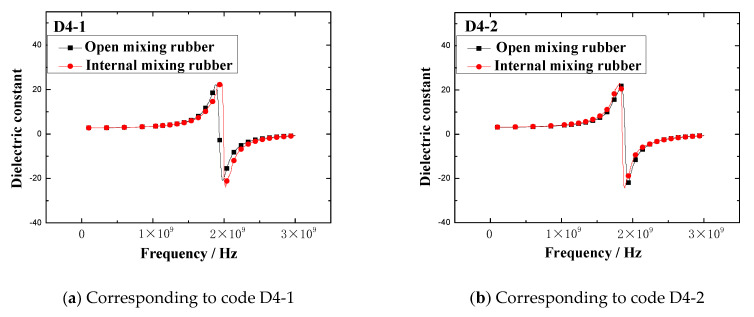

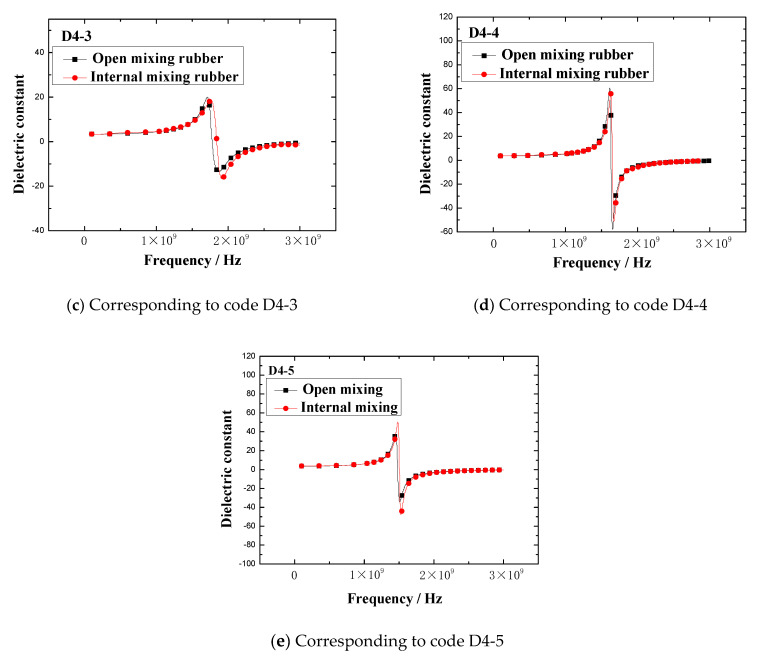
The dielectric constant vs. frequency plots of the rubber mixing.

The dielectric constant vs. frequency plots of open mixing rubber and internal mixing rubber are shown in Figure 5.

**Figure 6:**
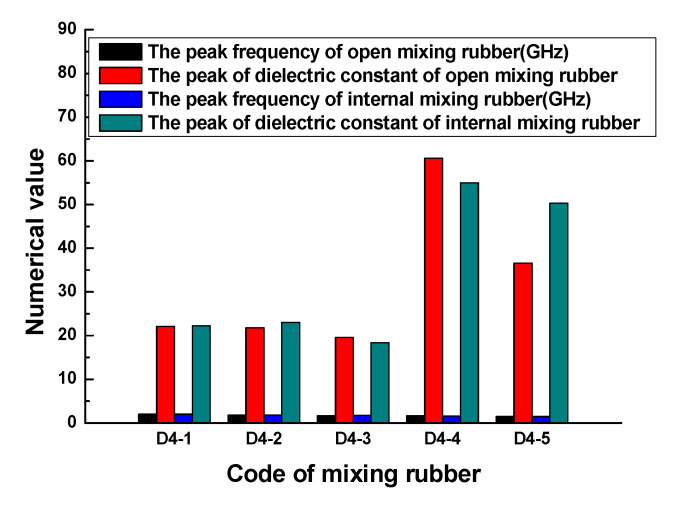
The peaks of the dielectric constant and frequency of the rubber samples.

Figure 5 indicates that no matter what kind of mixing method, the trend of the dielectric constant with the frequency is consistent. At lower frequencies, the dielectric constant gradually increased with the frequency, when the frequency increased to a certain value, the dielectric constant rapidly increased to a peak, then rapidly dropped to a valley value, and then, the dielectric constant gradually increased to a stable value with the frequency. The peak of the dielectric constant and the corresponding frequency are shown in Figure 6. The results showed that the peak and peak frequency of the dielectric constant were obviously affected by the mixing process. For the same formula, the peak dielectric constants of rubber samples obtained by different mixing methods had obvious differences. For rubber samples with codes D4-1, D4-2, D4-3, D4-4, and D4-5, the relative percentages of the peak dielectric constant difference were 0.9%, 5.2%, 6.0%, 9.2%, and 27.2%, respectively. At the same time, the frequencies corresponding to different peak dielectric constants were also significantly different. The relative percentages of the peak frequency difference were 1.0%, 1.6%, 2.3%, 1.8%, and 2.0%, respectively. 

**Figure 7:**
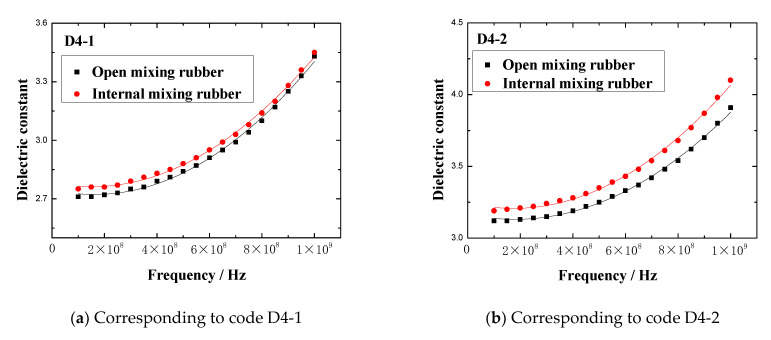

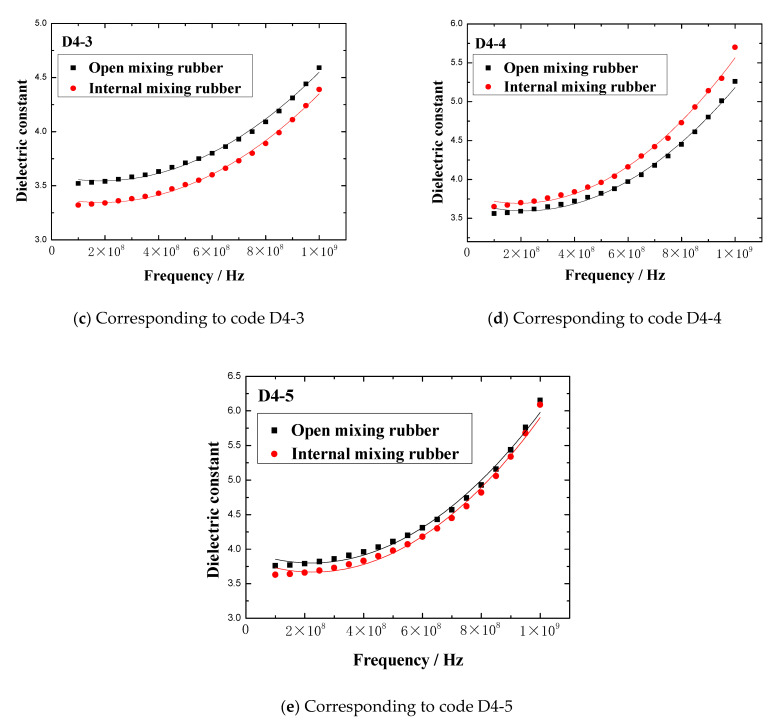
The dielectric constant vs. frequency plots of the rubber samples with different mixing process.

By observing Figure 5, it can be seen that the dielectric constants fluctuated significantly in the frequency range of 1 GHz to 2 GHz. In other frequency ranges, the dielectric constant was almost unaffected by the mixing process. In order to explore whether the dielectric constant was affected by the mixing process in other frequency ranges, the dielectric constants in the frequency range of 0.1 GHz to 1 GHz were selected as the research focus. In the frequency range of 0.1 GHz to 1 GHz, the dielectric constant vs. frequency plots of two kinds of rubber mixing are shown in Figure 7.

**Figure 8:**
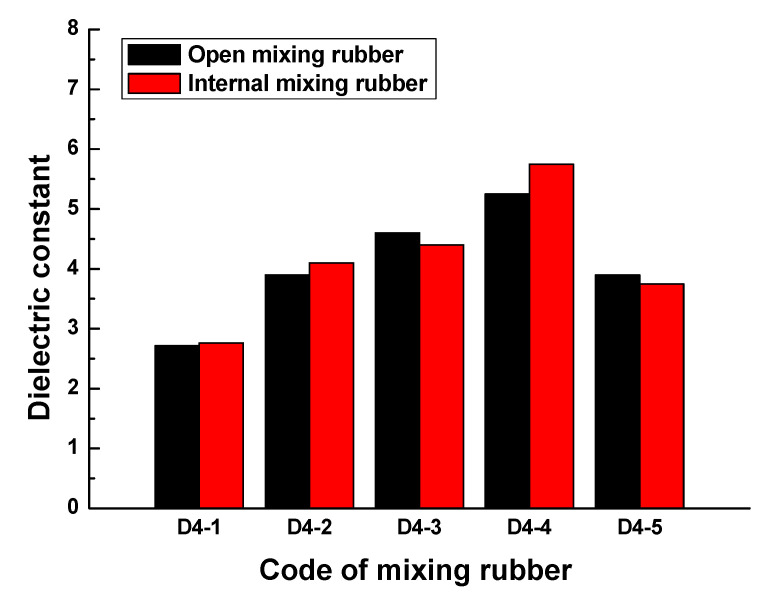
The maximum dielectric constants of the rubber samples obtained by different mixing processes.

Figure 7 indicates that the effect of the mixing process on the dielectric constant of mixing rubber was also remarkable in the frequency range of 0.1GHz to 1GHz. In the frequency range of 0.1GHz to 1GHz, the comparison of the maximum dielectric constants of rubber samples obtained by different mixing processes is shown in Figure 8. For rubber samples with codes D4-1, D4-2, D4-3, D4-4, and D4-5, the relative percentages of the maximum dielectric constant difference were 1.6%, 4.9%, 4.3%, 8.7%, and 3.8%, respectively. So, the effect of the mixing process on the dielectric properties of rubber materials cannot be ignored.

### 3.2. Effects of CB, ZnO, and SA

CB can not only be used as a rubber reinforcing agent but also can improve the microwave absorption properties of rubber materials. However, the absorbing properties of rubber materials are controlled by their dielectric properties. The research results in Section 3.1 also showed that the dielectric constant of rubber materials was affected by the CB content. Therefore, it is necessary to study the effect of the CB content on the dielectric properties of rubber materials.

The ultimate goal of preparing the mixing rubber is to vulcanize it into rubber products, so sulfur is essential as a vulcanizing agent. Based on formula one, formula two, formula three, and formula four, the same amount of S was added to the natural rubber in the process of preparing the rubber compounds. The codes for the rubber compounds containing S are listed in Table 6.

#### 3.2.1. Effects of CB, ZnO, and SA on the Dielectric Constant

**Figure 9:**
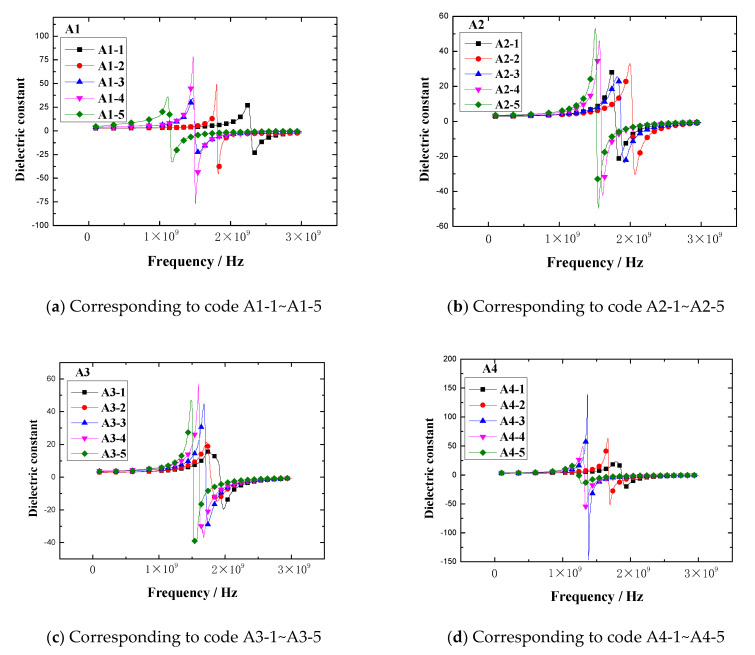
The dielectric constant vs. frequency plots of the rubber samples.

The dielectric constant vs. frequency plots of the rubber samples are shown in Figure 9.

**Figure 10:**
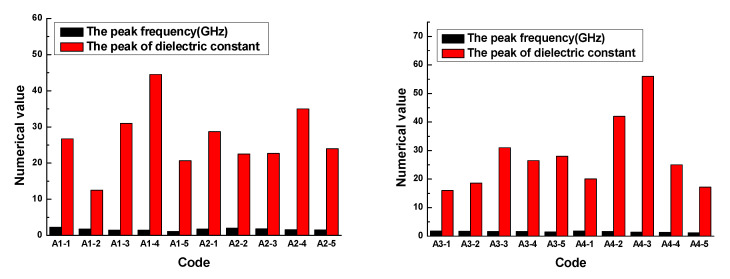
The peak and peak frequency of the dielectric constant of the rubber samples.

The results demonstrated that the dielectric constant of the rubber sample also changed with the frequency. The dielectric constant of each rubber sample also had a peak and valley value. The peak of the dielectric constant and the corresponding frequency are shown in Figure 10. Figure 10 shows that the dielectric constant of the rubber sample was significantly affected by the CB content. According to the grouping in Table 6, when other additives were added in the same amount, the maximum relative percentages of the dielectric constant difference of rubber samples with different CB contents were 71.9%, 35.7%, 48.4%, and 92.4%, respectively. The peak frequency decreased with the increase in CB content, that is, the higher the CB content, the lower the peak frequency. By observing and analyzing Figure 6, a similar influence law can also be obtained. Comparing the results of group two and group four, it can be seen that the dielectric constant of the rubber sample was also significantly affected by the ZnO content. When other additives were added in the same amount, the relative percentage of the dielectric constant difference of rubber samples with different ZnO contents reached 59.5%. The peak frequency of the rubber sample containing more ZnO was less than the peak frequency of the rubber sample containing less ZnO. The maximum relative percentage of peak frequency reached 24.7%. Comparing the results of group three and group four, it can be seen that the dielectric constant of the rubber sample was also affected by the SA content. When other additives were added in the same amount, the relative percentage of the dielectric constant difference of the rubber samples with different SA contents reached 55.7%. Contrary to the effect of ZnO content, the peak frequency of the rubber sample containing more SA was greater than the peak frequency of the rubber sample containing less SA. The maximum relative percentage of peak frequency reached 22.6%.

#### 3.2.2. Effects of CB, ZnO, and SA on the Loss Factor

**Figure 11:**
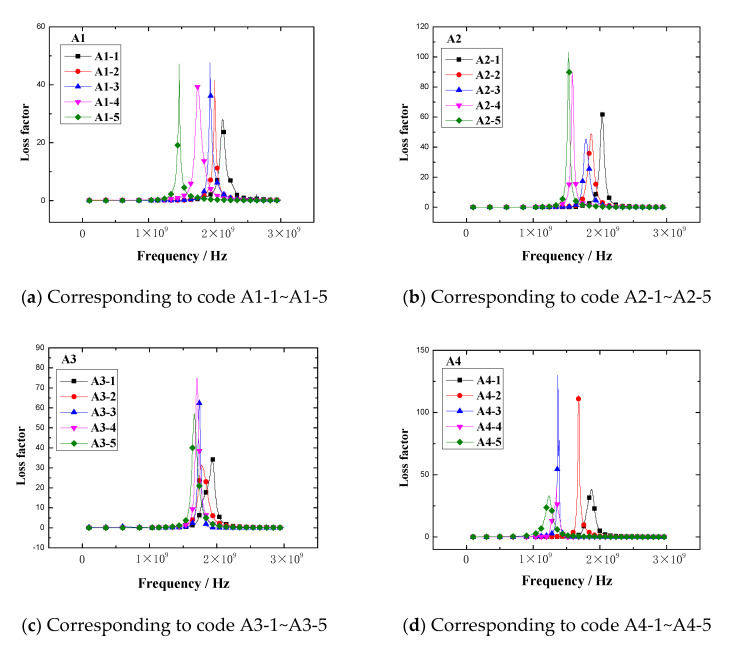
The loss factor vs. frequency plots of the rubber samples.

The loss factor vs. frequency plots of rubber samples are shown in Figure 11.

**Figure 12:**
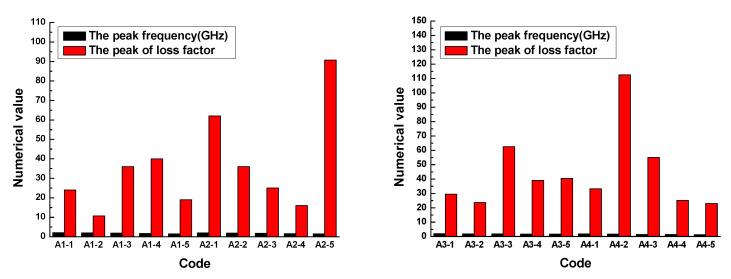
The peak and peak frequency of the loss factor of the rubber samples.

The results demonstrated that the loss factor of the rubber sample also changed with the frequency. The loss factor of each rubber sample also had a peak. The peak of the loss factor and the corresponding frequency are shown in Figure 12. Figure 12 shows that the loss factor of the rubber sample was also significantly affected by the CB content. According to the grouping in Table 6, when other additives were added in the same amount, the maximum relative percentages of the loss factor difference of rubber samples with different CB contents were 73.3%, 82.4%, 62.1%, and 79.6%, respectively. The peak frequency decreased with the increase in CB content, that is, the higher the CB content, the lower the peak frequency. Comparing the results of group two and group four, it was found that the loss factor of the rubber sample was also significantly affected by the ZnO content. When other additives were added in the same amount, the relative percentage of the loss factor difference of rubber samples with different ZnO contents reached 74.6%. Similarly, the peak frequency of the rubber sample containing more ZnO was less than the peak frequency of the rubber sample containing less ZnO. The maximum relative percentage of peak frequency reached 23.5%. Comparing the results of group three and group four, it was found that the loss factor of the rubber sample was also affected by the SA content. When other additives were added in the same amount, the relative percentage of the loss factor difference of rubber samples with different SA contents reached 78.9%. Contrary to the effect of ZnO content, the peak frequency of the rubber sample containing more SA was greater than the peak frequency of the rubber sample containing less SA. The maximum relative percentage of peak frequency reached 25.9%.

#### 3.2.3. Dielectric Parameters in the Frequency Range of 0.1 GHz–1 GHz

By observing Figure 9 and Figure 11, similarly, it was found that the dielectric constant and loss factor fluctuated significantly in the frequency range of 1GHz to 2GHz, however, the difference was not obvious in other frequency ranges. Therefore, the dielectric constant and loss factor in the frequency range of 0.1 GHz to 1 GHz were selected as the research focus to explore the effect of CB, ZnO, and SA.

**Figure 13:**
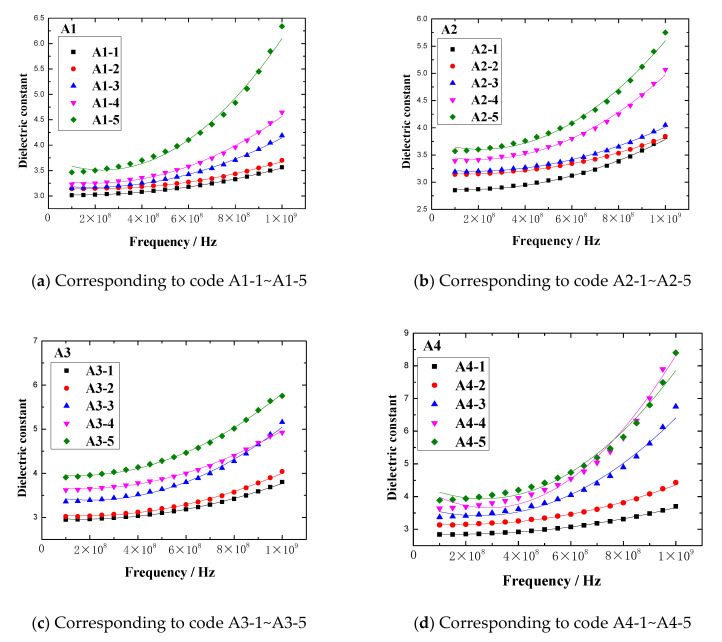
The dielectric constant vs. frequency plots of the rubber samples in the frequency range of 0.1–1 CHz.

**Figure 14:**
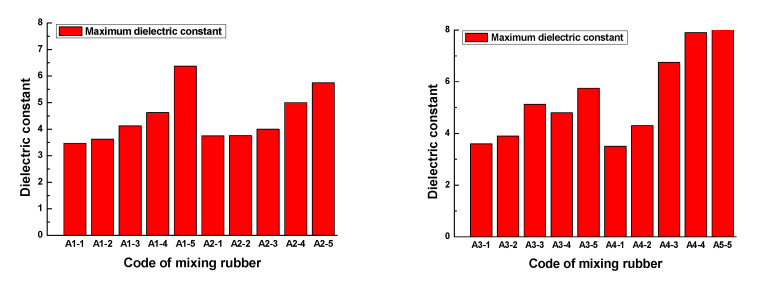
The maximum dielectric constant of the rubber sample.

In the frequency range of 0.1GHz to 1GHz, the dielectric constant vs. frequency plots of rubber sample are shown in Figure 13. The maximum dielectric constant of each rubber sample is shown in Figure 14.

Figure 13 and Figure 14 show that the dielectric constant of the rubber sample increased with increasing CB content. The increased percentages of the maximum dielectric constant were 83.7%, 53.3%, 59.7%, and 142.9%, respectively. Comparing Figure 13b,d, and combined with Figure 14, it can be seen that the dielectric constant increased with the increase in ZnO content, the increased percentage of the maximum dielectric constant reached 47.8%, and the higher the ZnO content, the higher the rate of dielectric constant increased with frequency. Especially when the CB content exceeded 15, this phenomenon became more obvious. However, comparing Figure 13c,d, combined with Figure 14, it can be seen that the dielectric constant decreased with the increase in SA content, the reduction percentage of the maximum dielectric constant reached 32.4%, and the lower the SA content, the higher the rate of dielectric constant increasing with the frequency.

**Figure 15:**
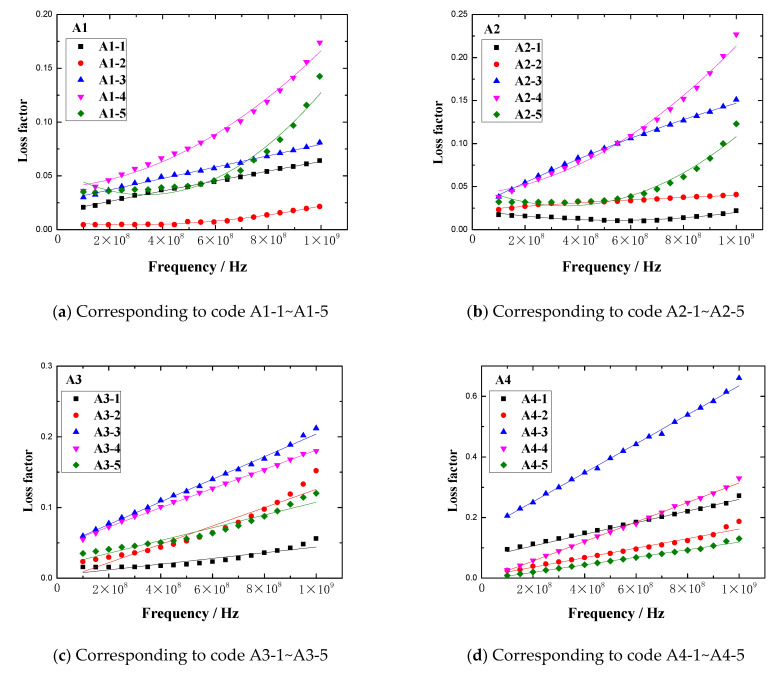
The loss factor vs. frequency plots of the rubber samples in the frequency range of 0.1–1 CHz.

**Figure 16:**
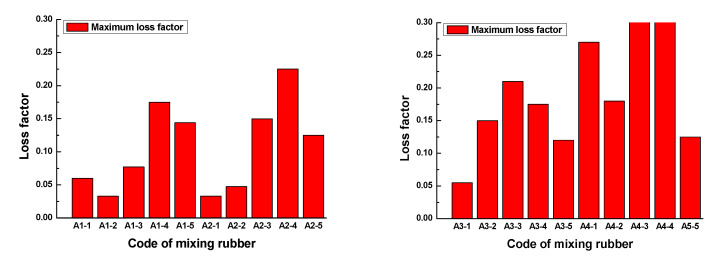
The maximum loss factor of the rubber samples.

In the frequency range of 0.1GHz to 1GHz, the loss factor vs. frequency plots of rubber samples are shown in Figure 15. The maximum loss factor of each rubber sample is shown in Figure 16.

Figure 15 and Figure 16 show that the loss factor of the rubber sample was significantly affected by the CB content in the frequency range of 0.1GHz–1GHz. When the CB content was 15 or 20, the loss factor had a maximum value. For the four groups of samples coded A1, A2, A3, and A4, the increased percentages of the maximum loss factor were 430.3%, 581.8%, 281.8%, and 420%, respectively. Similarly, the loss factor increased with the increase in ZnO content, the increased percentage of the maximum loss factor reached 188.9%. The loss factor decreased with the increase in SA content, the reduction percentage of the maximum loss factor reached 67.7%.

## 4. Conclusions

The dielectric constant and loss factor of the rubber sample vary depending on the frequency. No matter what kind of mixing method, the trend of the dielectric constant with the frequency is consistent. The effect of the mixing process on the dielectric constant of rubber samples is remarkable. For rubber samples prepared by two methods, open mixing and internal mixing, the relative percentages of the peak dielectric constant difference are 0.9%, 5.2%, 6.0%, 9.2%, and 27.2%, respectively. In addition, in the frequency range of 0.1GHz to 1GHz, the relative percentages of the maximum dielectric constant difference are 1.6%, 4.9%, 4.3%, 8.7%, and 3.8%, respectively. Hence, the effect of the mixing process on the dielectric properties of rubber materials cannot be ignored. 

The dielectric constant and loss factor fluctuate drastically in the frequency range of 1GHz to 2GHz. The dielectric constant has a peak and valley value, while the loss factor only has a peak. The peak frequency decreases with the increase in CB content or ZnO content, but increases with the increase in SA content. The dielectric constant of the rubber sample is significantly affected by the content of CB, ZnO, and SA. When other additives are added in the same amount, the maximum relative percentages of the dielectric constant difference of rubber samples with different CB contents are 71.9%, 35.7%, 48.4%, and 92.4%, respectively. The relative percentage of the dielectric constant difference of the rubber samples with different ZnO contents reached 59.5%. The relative percentage of the dielectric constant difference of the rubber samples with different SA contents reached 55.7%. The dielectric constant of the rubber sample increases with CB content or ZnO content, but decreases with the increase in SA content.

The loss factor of the rubber sample is also obviously affected by the content of CB, ZnO, and SA. When other additives are added in the same amount, the maximum relative percentages of the loss factor difference of the rubber samples with different CB contents are 73.3%, 82.4%, 62.1%, and 79.6%, respectively. The relative percentage of the loss factor difference of rubber samples with different ZnO contents reached 74.6%. The relative percentage of the loss factor difference of the rubber samples with different SA contents reached 78.9%. The loss factor increases with the increase in ZnO content but decreases with the increase in SA content.

## Figures and Tables

**Table 1:** The technical indicators of ZnO and CB.

ZnO	
Relative molecular mass	81.39
ZnO content	≥99.0%
Clarity test	Qualified
Free base	Qualified
Dilute sulfuric acid insoluble matter	≤0.01%
Chloride (Cl)	≤0.001%
Sulfur compounds (calculated as SO_4_)	≤0.01%
Nitrate (NO_3_)	≤0.003%
Mn	≤0.0005%
Fe	≤0.0005%
As	≤0.00005%
Pb	≤0.005%
Reduced potassium permanganate substance (calculated as O)	≤0.002%
Ammonium sulfide that does not precipitate (calculated as sulfate)	≤0.10%
**CB**	
Fixed carbon content of graphite powder	≥99.9%
Ash	≤0.36%
Moisture	≤0.36%
pH value	6.2
Mesh	5000

**Table 2:** The technology parameters of the broadband dielectric and impedance spectroscopy.

Frequency range/MHz	1–3
Impedance range/Ohm	0.01–10^5^
Capacitance range/F	10^−15^–1
Temperature range/℃	+20–+40
Temperature stability/℃	±0.2
Maximum heating and cooling rate/℃·min^−1^	50
Phase difference accuracy	2 × 10^−3^
Loss accuracy: tanδ	3 × 10^−5^
Measuring voltage/Vrms	0–3
DC bias/V	±40

**Table 3:** The rubber mixing formulas.

Raw Material	Mass/g									
	Formula 1					Formula 2				
	1-1	1-2	1-3	1-4	1-5	2-1	2-2	2-3	2-4	2-5
NR	100	100	100	100	100	100	100	100	100	100
ZnO	1	1	1	1	1	2	2	2	2	2
SA	1	1	1	1	1	2	2	2	2	2
CB	5	10	15	20	25	5	10	15	20	25
	Formula 3					Formula 4				
	3-1	3-2	3-3	3-4	3-5	4-1	4-2	4-3	4-4	4-5
NR	100	100	100	100	100	100	100	100	100	100
ZnO	3	3	3	3	3	3	3	3	3	3
SA	3	3	3	3	3	2	2	2	2	2
CB	5	10	15	20	25	5	10	15	20	25

**Table 4:** The mixing conditions.

The Open Mixing Conditions	
Roller speed	20 r/min
Mixing time	9–10 min
Roller temperature	≤50 °C
Roll distance of calendering sheet	1.8 mm
The Internal Mixing Conditions	
Rotating speed	90 r/min
Mixing time	10 min
Feed temperature	70 °C
Rubber discharging temperature	130–140 °C

**Table 5:** The codes of the rubber mixing.

Code	D4-1	D4-2	D4-3	D4-4	D4-5
NR	100	100	100	100	100
ZnO	3	3	3	3	3
SA	2	2	2	2	2
CB	5	10	15	20	25

**Table 6:** The codes for the rubber compounds containing S.

CB Content	5	10	15	20	25
Corresponding formula	Code				
Formula 1 (group one)	A1-1	A1-2	A1-3	A1-4	A1-5
Formula 2 (group two)	A2-1	A2-2	A2-3	A2-4	A2-5
Formula 3 (group three)	A3-1	A3-2	A3-3	A3-4	A3-5
Formula 4 (group four)	A4-1	A4-2	A4-3	A4-4	A4-5

## Data Availability

The data presented in this study are available on request from the corresponding author.
